# Fatal Acute Heart Failure in the Course of Macrophage Activation Syndrome: Case Report and Literature Review

**DOI:** 10.3390/jcm11144208

**Published:** 2022-07-20

**Authors:** Jakub Kuna, Grzegorz Chmielewski, Marcin Gruchała, Jolanta Szade, Mateusz Mikiewicz, Przemysław Ręcki, Magdalena Krajewska-Włodarczyk

**Affiliations:** 1 Department of Rheumatology, School of Medicine, Collegium Medicum, University of Warmia and Mazury, 10-900 Olsztyn, Poland; gchmielewski.gc@gmail.com; 2 First Department of Cardiology, Medical University of Gdańsk, 80-211 Gdańsk, Poland; mgruch@gumed.edu.pl (M.G.); precki@uck.pl (P.R.); 3 Department of Pathomorphology, Medical University of Gdańsk, 80-214 Gdańsk, Poland; jolanta.szade@gumed.edu.pl; 4 Department of Pathological Anatomy, Faculty of Veterinary Medicine, University of Warmia and Mazury in Olsztyn, Oczapowskiego St. 13, 10-719 Olsztyn, Poland; mateusz.mikiewicz@uwm.edu.pl

**Keywords:** macrophage activation syndrome, heart failure, myocarditis, systemic lupus erythematosus

## Abstract

Macrophage activation syndrome is a severe and potentially fatal condition in rheumatology. It can involve many different organs and systems, including the cardiovascular system, but heart failure due to its course is a relatively rare occurrence. In the following paper, we present a case of a young woman with newly diagnosed systemic lupus erythematosus who, in the span of two months, developed macrophage activation syndrome and acute heart failure, which caused her death. We analyze potential causes that may have led to that outcome, and present a brief review of the current literature concerning different macrophage groups in the heart and their potential involvement in the development of heart failure.

## 1. Introduction

Macrophage activation syndrome (MAS) is one of the relatively few diseases in rheumatology that requires urgent clinical intervention. It is characterized by a very diverse clinical course, which can cause acute failure in many organs and systems. MAS, currently classified among secondary hemophagocytic lymphohistiocytoses (sHLHs), occurs in adults mainly during systemic lupus erythematosus (SLE); however, the largest number of its cases have been recorded in patients with juvenile idiopathic arthritis (JIA/sJIA). It is also worth noting that patients with Still’s disease, rheumatoid arthritis, dermatomyositis, systemic scleroderma, Kawasaki disease, and other connective tissue diseases (including mixed connective tissue disease) are at higher risk of its occurrence. The rich and diverse symptomatology of this syndrome is dependent on which organs or systems are affected and the severity of the inflammation. The classification criteria focus on general symptoms (prolonged fever), irregularities in the hematopoietic system (hemophagocytosis—most often present in bone marrow, hyperferritinemia caused by the destruction of circulating RBCs, cytopenia in two or more cell lines, and splenomegaly), discrepancies in inflammation parameters (normal or decelerated ESR accompanied by an increased concentration of CRP), and sometimes a high concentration of transaminases (MAS study group criteria) or symptoms in the central nervous system (Ravelli’s criteria of 2011) [1]. CNS involvement may present in many different ways, such as confusion or somnolence, seizures, demyelinating lesions, or aseptic meningitis. Cardiac complications commonly occur as pericarditis and (mostly in adult patients with SLE) myocarditis. Renal failure and pancreatitis have also been reported in the medical literature. Lung involvement includes acute lung injury and, in rare cases, acute respiratory distress syndrome [2] or pulmonary arterial hypertension. Additionally, hepato- and splenomegaly are not uncommon occurrences; however, only the latter was included in the HLH-2004 guidelines [3].

The patient study presented below ran over the course of the nearly two months she was hospitalized, during which SLE was diagnosed. We observed the development of MAS and heart failure, which was the immediate cause of death.

## 2. Case Presentation

The patient, a 40-year-old veterinarian, noticed the initial symptoms of her condition roughly 10 months before her hospitalization: over that time, she had lost 15 kg of body weight and experienced periodic aches in her shoulder and knee joints. Laboratory examinations indicated a progressing normocytic anemia. She reported to the emergency ward with heightened symptoms, after which she was admitted to the internal medicine ward. Physical examination upon admission indicated tachycardia (120/min), a few scattered crepitations over the lungs, and the ECG showed a right axis deviation with no signs of myocardial ischemia. Laboratory examinations conducted during the patient’s stay at the ward indicated a significantly accelerated ESR (112 mm/h), dyselectrolitemia—in the form of hyponatremia and hyperkalemia at normal concentration of creatinine, heightened liver parameters—transaminases and GGTP, increased total protein concentration with decreased concentration of albumin, significant predominance of gamma globulins in electrophoresis, as well as high levels of ferritin and alkaline phosphatase. A 24 h urine sample contained 800 mg of protein. Testing for antibodies yielded positive results for IgM- and IgG-class antiphospholipid antibodies; pANCA antibodies; antinuclear antibodies at 1:3200 titer, including anti-SM, RNP/SM, and dsDNA; negative anticardiolipin antibodies; and lupus anticoagulant. Complement protein C3 was at a lowered level, while C4 was within norm. Bone marrow biopsy showed no deviations. Several pharyngeal swabs were used to culture strains of Klebsiella pneumoniae, then methicillin-resistant Staphylococcus aureus; subsequent microbiological tests were negative. No pathological microbes were cultured in blood and urine samples. The first point of focus was diagnosing the anemia and weight loss: abdominal ultrasonography indicated moderate splenomegaly (13 cm) and increased echogenicity of the liver. Endoscopy of the gastrointestinal tract was then performed: gastroscopy revealed signs of gastroduodenopathy (confirmed in histopathological examination) with a negative urease test; colonoscopy results were within norm. Due to persistent tachycardia and right axis deviation in the ECG tests, an echocardiogram was performed, which revealed no regional myocardial contraction dysfunction or widening of the heart chambers; no cardiac valvular abnormalities were detected with the exception of mild tricuspid and pulmonic regurgitation. In the pericardium, the presence of fluid was observed, ca. 7–8 mm thick. The ejection fraction was at 60%. In order to rule out pulmonary embolism, angio-tomography of the chest was performed: no embolism was detected; however, there were several lesions in the lung parenchyma, which were described as post-inflammatory changes. Medications included antibiotics based on the obtained antibiograms, as well as a beta-blocker.

Taking into account the patient’s medical history, laboratory tests, and imaging, systemic lupus erythematosus was diagnosed in accordance with the ACR and EULAR’s classification criteria of 2018. Thus, after four weeks of hospitalization in the internal ward, the patient was transferred to the rheumatology ward in order to determine further steps.

**Figure 1:**
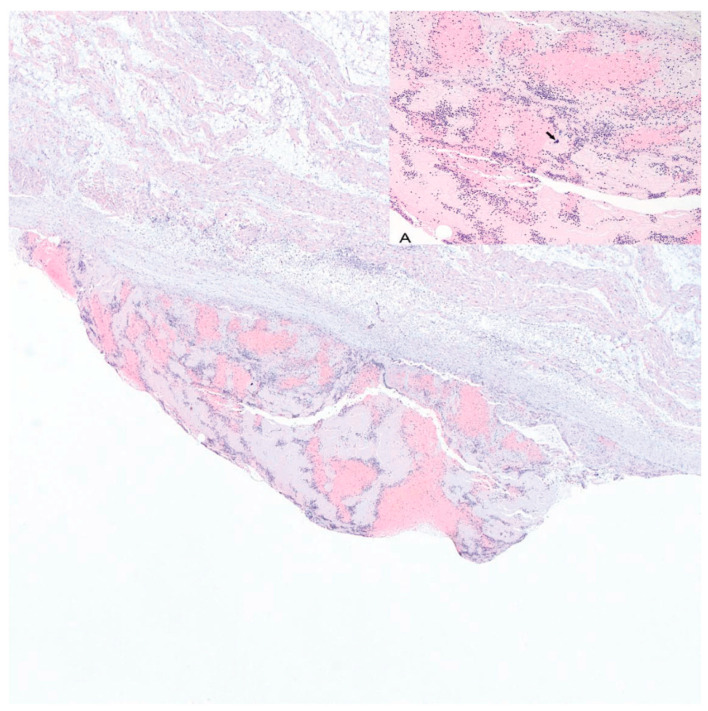
Right atrium, atrial surface of the tricuspital valve leaflet, 40-year-old woman. Vegetative endocarditis. HE, ×40. Inset (**A**): right atrium, atrial surface of the tricuspital valve leaflet, 40-year-old woman. Vegetative endocarditis. The vegetation consists of fibrin-platelet thrombi, germs colonies (arrow) and shows acute inflammation with neutrophils predominance. HE, ×100.

**Figure 2:**
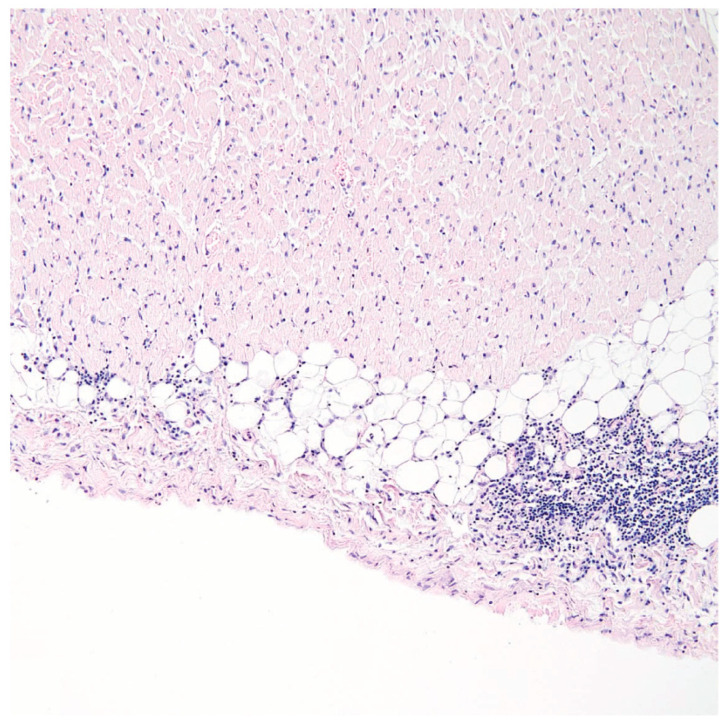
Epicardium, 40-year-old woman. Epicarditis with neutrophils and mononuclear cell infiltration. HE, ×100.

Examination upon admission revealed advancing anemia, as well as leukopenia and thrombocytopenia, persistent accelerated ESR, slightly increased CRP concentration, procalcitonin levels within norm, and negative cultures in blood, urine, and pharyngeal swabs. Creatinine concentration was increased, and the previously observed proteinuria continued (820 mg in a 24 h sample). Treatment began with intravenous pulses of 1 g methylprednisolone over 3 days, followed by a steroid administered orally; however, due to intensifying pancytopenia, intravenous doses were reintroduced, and mycophenolate mofetil was added to the medications. Control test results, especially the high concentrations of triglycerides, ferritin, and transaminases; lowered concentration of fibrinogen; as well as a single promyelocyte detected in the manual blood smear, pointed to the possible development of hemophagocytic syndrome in the patient. Mycophenolate mofetil was discontinued, while cyclosporin was simultaneously added, followed by human immunoglobulin. Two units of irradiated leukocyte-reduced red blood cells were transfused, and preventative doses of low-molecular-weight heparin were employed. The above treatments resulted in an improvement in the morphotic parameters of blood (normalized levels of leukocytes and platelets, and increased RBC parameters); reduced concentration of transaminases GGTP, triglycerides, and ferritin; fibrinogen returned to normal levels; and elimination of the proteinuria. Despite the improved laboratory test results in the first days following the immunoglobulin treatment, the general condition of the patient deteriorated, beginning with fatigue and increased swelling of the lower limbs, and followed by tachycardia, dry cough, auscultatory changes over the lung fields in the form of numerous crepitations, and dull percussive sounds in the lower fields of both lungs. ECG showed a persistent right axis deviation with no indication of acute ischemia symptoms. Control test results indicated a particularly high concentration of NT pro-BNP (above 35,000 pg/mL), and an increased but stable concentration of troponin T. CRP levels were slightly increased, while procalcitonin was within norm. Chest X-rays revealed areas of diffused interstitial consolidations in the lower lobes of both lungs and the perihilar area, as well as an enlargement of the heart. Abdominal ultrasonography indicated hepatosplenomegaly and small amounts of fluid in the peritoneal cavity. An urgent echocardiogram was performed, which revealed a general hypokinesis of the left ventricle, with the ejection fraction decreased to ca. 30%; TRPG was at 49 mmHg, and the fluid in the pericardium persisted at ca. 8 mm. Angio-tomography of the chest again showed no signs of pulmonary embolism but revealed a thickening of the intralobular septa and ground glass opacities with atelectatic areas at the base of both lungs. Furosemide, an ACE inhibitor, and a beta blocker were added to the medication. The patient was urgently transferred back to the internal ward, where, after a few days, she was transported to the intensive cardiological care ward of the First Department of Cardiology at the University Clinical Center in Gdańsk, with symptoms of acute heart failure. She died after several hours of reanimation. Post-mortem examination revealed symptoms of pulmonary edema with fluid in both pleural cavities, cardiac tamponade (150 mL of fluid in the pericardial sac), together with a widening of the left ventricle, fluid in the peritoneal cavity, nutmeg liver, and gallstones with no signs of inflammation. Histopathological examination revealed evidence of chronic endocarditis (Figure 1A), and epicarditis (Figure 2), but no signs of myocarditis.

We present selected laboratory findings and the left ventricular ejection fraction parameter from echocardiography in Table 1.

## 3. Discussion

This case study focuses on the final phase of a patient’s illness: the rapidly advancing heart failure and possible causes of its development. The most probable factors contributing to such a rapidly developing heart failure include: pulmonary embolism, acute coronary syndrome, cardiac tamponade, adverse drug effects, infection within the myocardium, acute tricuspid insufficiency, or inflammation in the course of macrophage activation syndrome.

Although an increased concentration of D-dimers (especially in the initial stage of the illness) and persistent right axis deviation in the ECG suggest pulmonary embolism, the repeated angio-tomographic chest examinations and the autopsy results do not confirm this diagnosis. Acute coronary syndrome can also be ruled out: the absence of ischemic changes in the ECG and regional myocardial contraction dysfunction in the echocardiograms, lack of a significant dynamic in repeated analyses of troponin T concentration (though its increased level is not unusual in the context of heart failure), and, most importantly, the lack of thrombotic material found in the coronary vessels during the autopsy all contradict such a diagnosis.

The presence of fluid in the patient’s pericardial sac was recorded with the initial echocardiogram: the thickness of the fluid layer at that time was 7–8 mm, and it remained largely unchanged in subsequent examinations, so it can be assumed that this corresponded with the volume of 150 mL measured during the autopsy. Of course, even a small amount of fluid in the pericardium may cause disruptions in the heart’s function; however, that requires a rapid increase in the fluid’s volume, which was not the case here.

Another possible cause of the failure that needs to be considered is an ongoing inflammation within the heart—a factor which we took into account with the first onset of symptoms of myocardial dysfunction, and which cannot be ignored in the final analysis. Repeated negative cultures of blood samples, the application of antibiotics adapted to the strains cultured in other samples, low concentrations of CRP, and normal or even lowered leukocyte count (together with lympho- and neutropenia) seem to contradict this hypothesis. On the other hand, histopathological examination of samples from the tricuspid valve revealed the presence of a contaminated thrombus with numerous neutrophils. Furthermore, a developing valve infection would explain the high value of the tricuspid regurgitation peak gradient (TRPG) parameter in the last echocardiogram performed on the patient, signifying acute valve regurgitation.

There are only a few studies concerning the epidemiology of MAS, particularly associated with SLE, but what can be surmised from all of the accounts is that myocardial invasion leading to acute heart failure is a rare occurrence. One of such analyses, documenting 362 cases of MAS in the course of sJIA, reported cardiovascular symptoms in 90 patients—which constitutes only 25% of cases; however, the majority of those cases involved pericardial invasion (57 patients, 16% of all cases), while heart failure was recorded in only 4 people (1.1% of all cases) [3]. Another study focused on 103 MAS episodes in 89 adult patients with SLE-reported cardiac complications in the form of pericarditis (23% of cases) and myocarditis (21%); however, heart failure was not singled out as a separate entity [2]. That said, we still think this mechanism to be a significant factor in the case of our patient.

Research from the last decade, conducted mainly on mice, paints macrophages residing in the heart not as a homogenous group of cells, but rather a morphologically and functionally diverse set of populations. It bears noting that the traditional division of macrophages into the pro-inflammatory M1 group and the anti-inflammatory M2 group has also been proved to be oversimplified: those groups are currently considered the extremes of a wide spectrum of possible macrophage “behaviors” and actual functions between these extremes. Differences in the expression of surface proteins and receptors for particular cytokines and chemokines allow for a certain level of structuring with regard to the functions of different macrophages. For macrophages residing in mouse hearts, these receptors are CCR2 (receptor for the C-C motif of the chemokine ligand 2 (CCL2), also known as the monocyte chemoattractant protein 1 (MCP1)) as well as class II MHC particles [4,5]. The first populations of macrophages can be observed in the epicardial layer during early fetal development; originating from the yolk sac, they are characterized by a low expression of the CCR2 and MHC-II receptors (and are hence labeled as CCR2− MHC-II low). The second group, appearing later in the prenatal period, are the endocardium-related macrophages, CCR2+ MHC-II low, originating from circulating fetal monocytes [6]. Additional groups appear and expire during various periods after birth. A mature heart houses three main groups of macrophages: CCR2− MHC-II low, CCR2− MHC-II high, and CCR2+ MHC-II high, as well as a population of CCR2+ MHC-II low [5]. There is one important study that attempted to determine the existence of analogous macrophage groups in the human heart—it must be noted that it involved the analysis of bioptates obtained during the implantation of a left ventricular assist device (LVAD), so it used material from patients already suffering from heart failure. Examination revealed populations of CCR2+ HLA-DR low (the human homologue of MHC-II), CCR2+ HLA-DR high, and CCR2− HLA-DR high [7].

The above classification of the populations, especially the division into the CCR2− and CCR2+ groups, is relevant to the analysis of their function in inflammatory processes. Tissue macrophages CCR2+ activate toward the M1 function and contribute to the recruitment of neutrophils and monocytes by producing proinflammatory chemokines and cytokines, further amplifying the inflammatory response. It has also been proven that CCR2+ macrophages that developed from monocytes and recruited this way exhibit an even higher proinflammatory activity compared with their tissue counterparts (they display a higher expression of proinflammatory chemokines and cytokines, and possess genes associated with pathological reconstruction of the myocardium). This process of recruiting monocytes/macrophages from circulating blood helps maintain the population of CCR2+ within the myocardium. CCR2− macrophages likely do not produce proinflammatory cytokines and chemokines, instead exhibiting a higher expression of growth factors (such as Igf1, Pdgfc, Hbegf, and Cyr61) and possessing genes responsible for myogenesis and DNA repair. Maintaining this population is wholly dependent on tissue reserves, as these macrophages only multiply locally and are not replenished from circulating monocytes. In the case of short-term inflammation (e.g., in response to acute myocardial ischemia), the population of the CCR2− macrophages usually remains relatively stable; however, an extended inflammatory process may lead to its significant depletion or even complete elimination, in which case they are replaced with CCR2+ cells. This results in subsequent inflammations being more severe, and contributes to a pathological reconstruction of the myocardium. It is postulated that this process explains the gradual deterioration of the left ventricle’s function with age [8,9].

It is assumed that there are three factors instrumental in the development of fully symptomatic MAS: a genetic abnormality (e.g., a mutation in the perforin coding gene PRF1—the first of many discovered to be responsible for MAS), a prolonged inflammation (as in the case of SLE or sJIA), and a trigger, such as an infection [10].

In the case of our patient, there are two possible causes of the acute heart failure. We suspect that despite the negative results of all blood cultures and the lack of significant deviations in the initial imaging examinations, from the first stages of the illness, we could have been dealing with a myocardial infection in the tricuspid valve area: the first echocardiogram, even though it did not reveal any vegetation on the valve’s flaps, did indicate its slight regurgitation—an incidental find that might have been disregarded in the later analysis, if not for the thrombus infiltrated by neutrophils discovered during the autopsy only in the environ of this valve. The developing infection led to a rapidly progressing tricuspid valve regurgitation, signified in the echocardiogram by a large increase in the TRPG parameter, that initially appeared as deficiencies in the function of first the right, then also the left ventricle.

It can be speculated that within a few weeks, the cytokine signal emitted by residual macrophages spurred the recruitment of circulating monocytes/CCR2+ macrophages. The prolonged inflammation, aggravated by the developing macrophage activation syndrome, may have led to the elimination or at least significant depletion of the local CCR2− macrophage population. Similar scenarios were described in mouse models of chronic myocarditis [5]. The macrophage constellation established this way, activated by circulating proinflammatory cytokines in the course of fully symptomatic MAS, may have led to an accelerated development of fully symptomatic acute heart failure [11].

The most commonly used pharmaceuticals in the treatment of MAS were applied in the medication of our patient—we administered high doses of methylprednisolone for the initial treatment, followed by cyclosporin and intravenous human immunoglobulin, but we did not employ biological pharmaceuticals. It is worth noting that some reports have indicated the usefulness of medications such as cyclophosphamide, etoposide, and biological agents such as anakinra, rituximab, and tocilizumab [2,3]. There is a single reported case of the beneficial effects of tocilizumab treatment in an adult patient with fully symptomatic MAS additionally complicated by myocarditis in the course of Still’s disease [12], but, as of yet, there are no guidelines or even deeper analyses of similar cases.

## 4. Conclusions

Macrophage activation syndrome is a severe and potentially fatal disease entity. Multiple organ failure may occur in its course; however, it rarely involves an invasion of the heart. This case study is an attempt to understand the processes potentially leading to this form of the illness, and highlights the need for a more detailed analysis of the disease pattern, which may allow us to predict its further course.

## Figures and Tables

**Table 1:** Laboratory and echocardiographic findings in the course of hospitalization.

Day after Admission	ESR (mm/h) [<12]	CRP (mg/L) [0–5]	ALT (U/L) [5–40]	Triglycerides (mg/dL) [35–135]	Ferritin (ng/mL) [13–150]	WBC (103/uL) [4–10]	HGB (g/dL) [12–15.5]	PLT (103/uL) [150–350]	Troponin T (ng/mL) [<0.014]	NT-pro BNP (pg/mL) [<125]	LVEF
1	112	13	257		830	5.47	8.8	306			
6											60%
12	137	7.3	45			3.21	7.8	252			
25	105	40	66			3.06	7.9	103			
33	92	9.4	106	501	1616	7.16	7.1	121			
38	98	22	97	230	589	3.87	9.3	226			
40	102	8.9	68	241	409	3.88	8.7	228			
42	65	27	55	193	332					32,561	
45									0.265		30%
47									0.244	>35,000	
48	40	24	192			7.86	9.4	266			
49											10%

## Data Availability

The analyzed data sets generated during the study are available from the corresponding author on reasonable request.
